# Potential Transcript-Based Biomarkers Predicting Clinical Outcomes of HPV-Positive Head and Neck Squamous Cell Carcinoma Patients

**DOI:** 10.3390/cells13131107

**Published:** 2024-06-26

**Authors:** J. Omar Muñoz-Bello, Sandra L. Romero-Córdoba, J. Noé García-Chávez, Claudia González-Espinosa, Elizabeth Langley, Marcela Lizano

**Affiliations:** 1 Unidad de Investigación Biomédica en Cáncer, Instituto Nacional de Cancerología, Mexico City 14080, Mexico; j.noe.garcia.c@gmail.com (J.N.G.-C.); langleyemx@gmail.com (E.L.); 2 Departamento de Medicina Genómica y Toxicología Ambiental, Instituto de Investigaciones Biomédicas, Universidad Nacional Autónoma de Mexico, Ciudad Universitaria, Mexico City 04510, Mexico; sromero@iibiomedicas.unam.mx; 3 Departamento de Bioquímica, Instituto Nacional de Ciencias Médicas y Nutrición Salvador Zubirán, Mexico City 14080, Mexico; 4 Unidad de Análisis Moleculares Don Vasco, Uruapan 60080, Mexico; 5 Departamento de Farmacobiología y Centro de Investigación sobre el Envejecimiento, Centro de Investigación y de Estudios Avanzados, Unidad Sede Sur, Mexico City 14330, Mexico; cgonzal@cinvestav.mx

**Keywords:** head and neck squamous cell carcinoma, human papillomavirus, biomarkers, transcripts, gene expression

## Abstract

Human papillomavirus (HPV)-positive Head and Neck Squamous Cell Carcinomas (HNSCC) comprise a particular cancer entity traditionally associated with better clinical outcomes. Around 25% of HNSCC are HPV positive, HPV16 being the most prevalent type. Nevertheless, close to 30% of the HPV-positive patients have an unfavorable prognosis, revealing that this type of tumor exhibits great heterogeneity leading to different clinical behaviors. Efforts have been made to identify RNA molecules with prognostic value associated with the clinical outcome of patients with HPV-positive HNSCC, with the aim of identifying patients at high risk of metastasis, disease recurrence, and poor survival, who would require closer clinical follow-up and timely intervention. Moreover, the molecular identification of those HPV-positive HNSCC patients with good prognosis will allow the implementation of de-escalating therapeutic strategies, aiming to reduce side effects, resulting in a better quality of life. This review compiles a series of recent studies addressing different methodological and conceptual approaches aimed at searching for potential gene expression-based biomarkers associated with the prognosis of patients with HPV-positive HNSCC.

## 1. Introduction

Head and Neck Squamous Cell Carcinomas (HNSCC) are part of a set of tumors affecting the upper part of the aerodigestive tract. These neoplasms develop from the epithelium of the mucosa of the oral cavity, lip, larynx, nasopharynx, oropharynx, hypopharynx and salivary glands [1]. In 2020, a total of 931,931 new cases and 467,125 deaths were estimated worldwide, ranking sixth and seventh in mortality and incidence, respectively, among all types of cancer [2]. Most of the head and neck tumors are histologically classified as squamous cell carcinomas, representing more than 90% of cases [3,4]. The incidence of head and neck tumors continues to rise worldwide, with an estimated increase of 30% annually by 2030 [4].

Several risk factors proposed to be associated with the development of HNSCC include alcohol [5,6] and tobacco [7] consumption. Interestingly, alcohol together with tobacco significantly increases the risk of developing this type of neoplasia [8]. In addition, it has been described that chewing betel nut, a common practice in Eastern countries, increases the risk of developing head and neck tumors, regardless of alcohol and tobacco [9]. Furthermore, biological agents such as viral infections, particularly Human Papillomavirus (HPV), have been described as etiological agents for the establishment of HNSCC [10]. The average age of patients at the time of diagnosis of HPV-negative HNSCC is approximately 66 years, while in HPV-positive tumors, the incidence in adults under 53 years of age has increased in recent years, particularly in the oropharynx, which is the anatomical subsite most frequently associated with HPV infection [1,11]. Furthermore, in individuals who, in addition to being smokers and alcohol drinkers, are HPV positive, there is a combined effect that considerably increases the risk of HNSCC, particularly in oral and oropharyngeal cancer [12,13]. For instance, reports show that the risk of oral cancer in people with HPV infection who have ever been smokers or alcohol drinkers is 6.8 and 4.8, respectively; while in those without HPV infection who have smoked or consumed alcohol, the risk is about 1.2. and 1.8, respectively. Particularly in HPV-positive oropharyngeal cancer, the risk in those individuals who ever smoked and consumed alcohol increases substantially to 13 and 10, respectively [12].

The overall prevalence of HPV in HNSCC is close to 25% [14,15,16], which differs depending on the anatomical subsites. For instance, it has been reported that HPV is present in 24.9% of oropharyngeal tumors, while only in 4.4% and 5.7% of oral and larynx cancers, respectively [17]. HNSCC has traditionally been considered a disease of older people; however, in recent years, a rising trend in the incidence of oropharyngeal and oral cancers has been reported in younger population (<45 years) worldwide [18]. The increased incidence of oropharyngeal tumors in the young population is partially explained by changes in the sexual behaviors of this population, where HPV infection would be highly relevant [19]. Moreover, in the United States, the incidence of HPV-positive oropharyngeal tumors increased by 225%, while that of HPV-negative cancers decreased by 50% between 1988 and 2004 [20]. Concordantly, a 2.9-fold increase in HPV-positive tonsillar cancer was observed in a Swedish population from 1970 to 2002 [21]. It was estimated that, if the incidence trend continues, the number of HPV-positive oropharyngeal cancer cases will exceed those with cervical cancer in the following years [20].

Among HPV-positive HNSCCs, most have the presence of HPV-16 and constitute approximately 90% of the cases [16], while a low proportion of tumors are positive for other high-risk viral types, including HPV-18, -31, -33, -45, -52, and -58. Particularly, in HPV-positive tumors of the oropharynx, oral cavity and larynx, HPV-16 has a prevalence of 83.4%, 70.9% and 50.8%, respectively [17]. All these viral types are considered in the nonavalent vaccine, which is expected to be highly effective for the prevention of HPV-positive HNSCC. A recent study evaluated the estimated incidence of oropharyngeal cancer, considering the HPV vaccination rate in the period between 2018 and 2045, and found that the incidence of HNSCC will be reduced in the young population (36–55 years), but will increase among older individuals, who have not yet been vaccinated and continue to be at high risk for developing HNSCC. Furthermore, it is expected that there will be no impact on the incidence of HPV in the global oropharyngeal cancer population [22].

Due to the intrinsic features of HNSCC tumors, it has been postulated that HPV-positive and -negative tumors have separate identities and differ in their molecular biology and clinical evolution. Furthermore, although several therapeutic strategies based on HPV status are currently being investigated at the clinical and preclinical levels, there are still no differences in treatments for HPV-positive and negative HNSCC patients [23]. It is well-described that patients with HPV-positive HNSCC have better Overall Survival (OS) compared to those that are HPV-negative [24], with 5-year survival rates of 75% to 80%, since HPV-positive HNSCC tumors show a better response to chemo- and radiotherapy [25,26]. Accordingly, median survival was significantly longer in HPV-positive compared with HPV-negative oropharyngeal tumors (131 vs. 20 months) [17]. Furthermore, given that HPV-positive HNSCC has better survival, it is important to analyze the possibility of de-intensifying current treatments, which would have an impact on improving quality of life and patient survival.

Moreover, it has been demonstrated that in patients with oropharyngeal cancer positive for p16, a surrogate marker for the presence of HPV, 3-year Progression-Free Survival (PFS) was higher compared to the group of p16-negative patients (72.8% vs. 49.2%), showing that patients with HPV-negative tumors have a higher probability of disease recurrence [27]. Furthermore, recurrence after treatment in patients with HPV-positive oropharyngeal cancer has been found to be less common when compared to patients with HPV-negative tumors. However, the five-year OS of patients with HPV-positive oropharyngeal cancer who develop recurrent disease is approximately 32% [28]. The incidence of HPV-positive HNSCC, particularly in oropharyngeal cancer, has been growing in recent years and, consequently, so has the number of cases of recurrent disease; therefore, there is a need to understand the molecular biology, clinical presentation, and potential treatments for recurrent disease in HPV-positive tumors. It is essential to identify molecules with prognostic value that allow the stratification of patients with high risk of disease recurrence and low survival, with the aim that patients with HPV-positive HNSCC can be beneficiaries of closer clinical follow-up and timely intervention, which will result in an improvement in quality of life.

## 2. Human Papillomavirus as a Determining Factor in the Establishment of HNSCC

HPV commonly infects the tonsillar crypt of the oropharynx, where the cells resemble those of the cervical squamocolumnar junction, which are organized into a discontinuous monolayer epithelium that is more susceptible to malignant transformation than cells of the oral cavity [26]. The productive viral cycle of HPV is closely related to the terminal differentiation program of keratinocytes in stratified mucosal epithelia; however, the interruption of this cycle promotes the appearance of persistent infection and the establishment of carcinogenesis [29]. The tonsillar crypts are a highly specialized and immune-privileged lymphoepithelial tissue, being densely infiltrated by lymphocytes. When HPV enters the basal cells of this tissue, it can block the activity of virus-specific T cells, facilitating immune evasion during the initial HPV infection [30]. Moreover, tonsillar crypts constitute a highly invaginated tissue and serve as a natural host for microorganisms. Cells at this site commonly express programmed cell death-1 ligand 1 (PD-L1), which is responsible for evasion of the immune response by binding to programmed cell death 1 (PD-1) receptors contained on immune cells. Therefore, HPV-infected tonsil cells can be recognized as belonging to the organism and are not eliminated, escaping immune surveillance, and allowing HPV infection to persist [26].

According to the papillomavirus episteme portal [31], more than 200 viral types capable of infecting the skin and mucosal epithelium have currently been described. Around 40 types of HPV infect the anogenital region, and half of them are associated with the establishment of several types of cancer such as cervix, anus, penis, vagina, vulva, and head and neck tumors [32]. Due to their oncogenic potential, HPVs have been classified into high risk (HR-HPV) and low risk (LR-HPV). Hitherto, HPV-16, -18, -31, -33, -35, -39, -45, -51, -52, -56, -58, -59, -68, -26, -53, -66, -67, -70, -73, -82, -30, -34, -69, -85 and -97, are considered to be HR-HPV types [33], with HPV-16 being the most prevalent in head and neck tumors, followed by HPV-18, in 86% and 27% of HPV-positive cases, respectively [34]. Additionally, coinfection of these two most prevalent viral types has been reported in 12% of the cases [34]. Most infections are transient, do not cause any symptoms or disease and disappear between 12 and 24 months after infection, being rapidly eliminated by the immune system. However, if such infections persist, the risk of progressing to cancer increases considerably. It has been shown that a determining event in viral oncogenesis is the integration of the HPV genome into the cellular genome, triggering the sustained expression of viral oncoproteins, E6 and E7, which participate in the neoplastic transformation of infected cells. However, it has been reported that E6 and E7 overexpression can also occur in the absence of viral genome integration through epigenetic changes in HPV transcription regulatory sequences [35]. The role of E6 and E7 oncoproteins in the establishment and maintenance of the tumor phenotype have been extensively described. It is known that E6 binds and degrades p53, promoting the accumulation of mutations and the inhibition of apoptosis, while E7 promotes the continuity of the cell cycle, through the binding and degradation of pRb [29]. Furthermore, HPV oncoproteins have been found to alter the expression profiles of transcripts, including messenger RNAs (mRNAs), microRNAs (miRNAs), and long non-coding RNAs (lncRNAs), in HNSCC-derived cell lines and HNSCC tumors, which are associated with the promotion of cancerous cellular processes [36,37,38]. The effects induced by viral oncoproteins together with alterations in the cellular genome can lead to cellular transformation towards malignancy, allowing the acquisition of hallmarks of cancer [29].

## 3. Proposed Transcript-Based Prognostic Biomarkers for HPV-Positive HNSCC

Based on the molecular biology of HPV-positive HNSCC, several molecules have been proposed with potential use as prognostic biomarkers to allow for the identification of patients who are at risk of having an unfavorable clinical outcome. In addition, analyzing the possibility of stratifying patients with potential benefits from the de-escalation of current treatments could lead to a reduction in side effects and an improvement in the quality of life of patients with HNSCC. A prognostic biomarker reports a probable cancer outcome, such as survival, recurrence and progression, regardless of the treatment that the patient received, thus reflecting the underlying biology and natural history of the disease [39]. In this sense, several biomolecules such as RNAs have been investigated as possible biomarkers in cancer related to HPV [40]. Currently, with the use of bioinformatics tools, open-access database repositories and more sophisticated and precise computational approaches, new innovative and promising prognostic biomarkers have been identified in several types of cancers based on the expression of coding or non-coding transcripts [41]. The most analyzed transcripts are mRNAs, miRNAs and lncRNAs. The mRNA is a single-stranded protein-coding RNA sequence, while miRNAs are small non-coding single-stranded RNA sequences of 18 to 24 nucleotides in length, which post-transcriptionally regulate gene expression by binding to the 3′ UTRs (untranslated regions), promoting mRNA degradation or preventing translation, which downregulates gene expression. Other non-coding RNAs are the lncRNAs with a sequence length of more than 200 nucleotides, which bind to proteins and nucleic acids to control cellular processes such as post-transcriptional regulation of mRNA, molecular signaling, scaffolding, and transcription, among others. Furthermore, lncRNAs act as molecular decoys by sponging miRNAs and preventing them from interacting with their target mRNAs [42]. Recent studies evaluating the potential use of transcripts as prognostic biomarkers in HPV-positive HNSCC are described in this review and summarized in Table 1. Such studies analyze transcriptomic data, either from public repositories such as The Cancer Genome Atlas (TCGA) and Gene Expression Omnibus (GEO) or those generated in independent cohorts using technologies such as next-generation sequencing. Given the heterogeneity of studies that define prognostic biomarkers in HPV-positive HNSCC, it is difficult to organize the results and compare them with each other. This heterogeneity includes differences in the workflow of bioinformatics analysis and in the methods used to identify HPV positivity, including the use of PCR, sequencing, in situ hybridization or p16 positivity. In addition, an important limitation is the small number of HPV-positive samples available in databases containing clinical information. Accordingly, in this review, we endeavor to recapitulate recent studies in HPV-positive head and neck tumors focused on identifying prognostic biomarkers based on candidate transcripts clearly associated with the clinical outcome of patients, regardless of the treatment received, including OS, PFS, Metastasis Free Survival (MFS), Disease-Free Survival (DFS) and Relapse-Free Survival (RFS). Therefore, we only present transcriptomic studies of tumor samples from human patients that provide evidence for the potential use of expression-level-based prognostic biomarkers in HPV-positive tumors.

### 3.1. lncRNAs

Recently, an expression profile of long non-coding RNAs (lncRNAs) was analyzed using transcriptomic data from a cohort of HNSCC patients from the TCGA database. The lncRNAs, *CDKN2B-AS1*, *TTTY14*, *TTTY15*, and *PRINS* were found over-expressed, while *MEG3* and *H19* appeared under-expressed in HPV-positive tumors. OS and DFS were significantly increased in patients with HPV-positive HNSCC with high *PRINS* expression, which was not influenced by clinicopathological features. Interestingly, high *PRINS* expression increased OS in patients with HNSCC regardless of HPV status. In addition, HPV-positive tumors with augmented *PRINS* expression are highly infiltrated by immune cells where the OS of patients with these tumors is significantly increased [43].

### 3.2. mRNAs

In clinical practice, there is a need to identify low-risk patients who can receive lower doses of treatment. As an approach, a clinically translatable immunological classification tool based on the abundance of three transcripts associated with OS and DFS was evaluated to find a molecular signature to identify patients with HPV-positive oropharyngeal squamous cell carcinoma (OPSCC) who may be candidates for lower therapy. RNAseq profiles of HPV-positive OPSCC samples were evaluated, with 16 cases showing some type of recurrence (local, regional and distal), and the tumor microenvironment (TME) was characterized by inferring the abundance of different cell populations using transcriptomic profiling. Tumors were categorized as immune-rich, immune-deserted and mixed, according to the UWO3 risk score, which is a signature based on the abundance of *CD3E*, *IRF4* and *ZAP70* transcripts. Patients with immune-rich environments were reported to have better DFS and OS compared to the desert immune group. These data provide evidence that the UWO3 score is a prognostic tool that can improve risk stratification of patients with HPV-positive OPSCC. Importantly, its utility has been demonstrated in real clinical scenarios [44].

Another study compared the transcriptional profile of 33 HPV-positive and 79 HPV-negative tumors from a TCGA cohort, resulting in 350 differentially expressed genes [45]. In addition, a weighted gene coexpression network analysis (WGCNA) identified 72 core genes related to HPV status that are associated with protein binding and mismatch repair pathways. Both approaches were combined, and 65 of the resulting transcripts were found to be associated with survival in HPV-positive tumors. In addition, an HPV-related prognostic model was obtained, consisting of eight genes: *Clorf105*, *CGA*, *CHRNA2*, *CRIP3*, *CTAG2*, *ENPP6*, *NEFH* and *RNF212*, which was associated with shorter survival in patients with HPV-positive HNSCC, both in the TCGA group of patients with HPV-positive tumors and in a validation cohort (GSE65858) [45].

A different approach to assessing the diversity of OPSCC-positive tumors evaluated the RNA-seq of eight samples, finding two molecular clusters based on differential expression of 148 genes. Cluster 1 was associated with metastatic recurrence at 18 months and death within 3 years, whereas cluster 2 had delayed metastasis and prolonged survival. The good prognostic cluster 2 was characterized by overexpression of *S100A7*, *S100A9*, *SPRR1A*, *SPRR1B*, *SPRR3*, *KRT6A*, *KRT6B* and *SERPINB1* and under-expression of *THBS4*, which are regulated by the transcription factor DNp63. In another cohort of 71 patients with HPV-positive OPSCC, the DNp63 protein level allowed dividing the cohort into high- and low-protein tumor groups, and *S100A9*, *SERPINB1* and *SPRR1A* genes were found to be highly expressed in the high DNp63 expression group, while *THBS4* is highly expressed in the low DNp63 group. In addition, *S100A9*, *SERPINB1* and *SPRR1A* genes were observed to be significantly more expressed in tumors without metastatic spread at 3 years, while *THBS4* expression was significantly lower. It was also observed that patients with low *S100A9* and high *THBS4* levels had an increased risk of metastatic progression, similar to those with low DNp63 levels. Taken together, these results show that both *S100A9*/*THBS4* and DNp63 expression define a molecular signature that influences the prognosis of patients with HPV-positive OPSCC [46].

One more study analyzed the expression of *TP53*, *NRF2*, *KEAP* and *NQO1* genes in patients from TCGA with HPV-16-positive HNSCC. By the mean expression value, patients were classified into the upper and lower quartiles and correlated with OS. Low expression of *NRF2*, *TP53* and *NQO1* genes and high expression of the *KEAP1* gene were significantly associated with better survival of patients with HPV-positive HNSCC [47].

Transcriptomic characterization of TCGA tumors comparing 409 patients with HPV-negative HNSCC vs. 66 HPV-16-positive patients identified 312 proteins with differential interactions. Using network topology, 19 modules were identified that could be used as potential biomarkers of HPV-positive and HPV-negative tumors based on the coordinated action of a group of biological entities. Seven of the modules (m*CBX5*, m*CDKN2A*, m*MCM5*, m*MCM6*, m*RBBP7*, m*TMPO*, and m*VCAM1*) function as specific prognostic biomarkers for HPV-16-positive HNSCC cancer with a high impact on overall patient survival. In concordance, the m*CDKN2A*, m*MCM5*, and m*VCAM1* modules were validated in an independent cohort (GSE65858), showing remarkable prognostic performance in HPV-16-positive samples [48].

*ZNF540* gene expression allowed to divide 60 HPV-positive HNSCC tumor samples into high- and low-expression groups (GSE65858). Significantly better OS was observed in patients with high *ZNF540* expression compared to the low expression group, with a median survival of 1249 days vs. 933 days, respectively [49].

In an approach to understand the relationship between transcriptional profiling and immune infiltration, the differential expression profiles of seven datasets (GSE65858, GSE117973, GSE55545, GSE40774, GSE39366, GSE6791, GSE55543) were analyzed in 167 HPV-positive and 574 HPV-negative HNSCC samples, identifying 52 differentially expressed genes in HPV-positive tumors, of which those with some association with inhibition of tumor progression and OS in a TCGA cohort of HNSCC were prioritized. High expression of the *FDCSP*, *NEFH* and *FAM3B* genes was associated with improved OS in HPV-positive HNSCs. Since the *FDCSP* gene is known to be closely related to the immune function of tumor tissue, immune infiltration of tumors was analyzed and correlated with the level of *FDCSP* expression (high and low). HPV-positive samples with high expression of *FDCSP* are enriched in follicular helper T, memory B cells, CD8+ T cells, memory CD4+ cells and macrophages. It was also observed that elevated *FDCSP* levels and CD8+ T cell abundance were associated with better prognosis in HPV-positive HNSCC with increasing OS [50].

A molecular characterization with TCGA transcriptomic data [51], which compared 26 HPV-p16-positive HNSCC samples, 71 HPV-p16-negative HNSCC samples and 44 adjacent normal control samples identified 102 deregulated lncRNAs, 196 deregulated miRNAs and 2282 deregulated mRNAs in the p16-positive group compared with adjacent normal tissue samples, of which 9 lncRNAs, 31 miRNAs and 167 mRNAs were significantly associated with patients’ OS. High expression of lncRNA *U62317.3*, low expression of hsa-miR-375 and high expression of mRNA *KLHDC7B* were strongly associated with improved OS in p16-positive HNSCC patients. The interaction of these transcripts was subsequently analyzed by competing endogenous RNA networks (ceRNAs) where 5 lncRNA nodes, 16 miRNA nodes, 66 mRNA nodes and 162 edges were identified. Among them, three mRNAs (*PDLIM5*, *USP25* and *SLMAP*) were identified within the ceRNA cluster in the p16-positive HNSCC, which were associated with OS [51].

A transcriptomic analysis of 414 HNSCC samples from a Mexican cohort, of which 17.5% were HPV and p16 positive, while 65.3% were HPV and p16 negative, identified 98 differentially expressed, 16 overexpressed and 82 under-expressed genes. Transcriptional profiling was validated in a TCGA cohort composed of 39 HPV-positive and 75 HPV-negative cases, validating 51 of the deregulated genes. Finally, elevated expression of *GJB2* and *SLC25A39* genes was associated with poor survival, exclusively in HPV-positive patients [52].

Continuing the characterization of tumor vs. non-tumor tissue, the *DOCK8* gene was found to be under-expressed in HNSCC tumor samples based on Oncomine data. When grouping HNSCC samples according to HPV status using TCGA mRNA-seq data, higher *DOCK8* expression was observed in HPV-positive HNSCC. In this same cohort, grouping 38 patients with HPV-positive HNSCC by the mean of the *DOCK8* expression value showed that higher *DOCK8* expression had a significantly more favorable OS compared to patients with lower expression, a phenotype only observed in patients with HPV-positive HNSCC. Similar results were obtained when DOCK8 expression was assessed in a cohort of 270 HNSCC patients with HPV status information (GSE65858). Seventy-three HPV-positive HNSCC patients in the cohort were separated according to high and low *DOCK8* expression and as expected, higher *DOCK8* expression in HPV-positive HNSCC patients was significantly associated with favorable OS and PFS [53].

In a study where 197 cases of OPSCC (167 HPV-positive and 30 HPV-negative cases) were analyzed by RNA sequencing, 1436 protein-coding genes associated with OS, whose prognostic value is independent of treatment, were found. The most promising gene biomarkers were selected, generating a signature composed of 60 genes associated with survival in patients with OPSCC. Twenty-six genes with high expression (*RASSF8*, *LGALSL*, *TRIB3*, *FAM106A*, *MICAL3*, *STIP1*, *ZNF146*, *ZNF284*, *TPST1*, *PSMG3*, *CPEB2*, *SH3D21*, *CNOT2*, *ENTPD6*, *PPIAL4C*, *SARS*, *SULT1E1*, *EAF1*, *LOC100128108*, *PLPPR2*, *OTUD4*, *MUL1*, *BCL2L13*, *GUCY1B1*, *MRPL45* and *GALK1*) were associated with poor survival; while elevated expression of 34 genes (*RSU1*, *AVPI1*, *PDPR*, *ALKBH6*, *HCK*, *ATP6V1A*, *TAF5L*, *BUD13*, *TNFRSF6B*, *ACOT11*, *RNF167*, *ORAI1*, *CRYBG3*, *HDDC3*, *CREB3L4*, *TMEM246*, *PEX16*, *HAUS1*, *NUP214*, *AURKB*, *OGN*, *FBXO41*, *SLFN13*, *CXCL13*, *COMMD3*, *FOXRED2*, *FPGS*, *UCP2*, *GLUL*, *LYN*, *MEI1*, *CYBA*, *NUP210* and *ARHGAP4*) was associated with favorable outcomes. Patients were grouped according to risk score into high and low. Survival analysis revealed that the risk of death of high-risk patients was significantly higher than that of low-risk OPSCC patients. When this 60-gene signature was analyzed only in patients with HPV-positive OPSCC, it was observed that the group of patients with the low-risk score had significantly superior OS, RFS and MFS. Similar data were obtained when analyzing this signature in a validation cohort, composed of 93 HPV-positive OPSCC cases, in which a low-risk 60-gene signature score predicted a significant increase in OS, RFS and MFS [54].

In a strategy guided by altered metabolic pathways between HPV-positive and HPV-negative HNSCC tumors from transcriptomic data obtained from TCGA, 229 genes were found that allowed grouping patients into high- and low-expression in 74 HPV-positive patients. Decreased expression of metabolism-related genes, in particular *SDHC*, *COX7A1*, *COX16*, *COX17*, *ELOVL6*, *GOT2* and *SLC16A2*, was significantly associated with improved survival in HPV-positive patients. In addition, increased OS was observed in HPV-positive patients when comparing the low expression of *COX16* and *COX17* genes with the high expression group. Similar results were obtained when analyzing the *COX16*/*SLC16A2* and *COX17*/*SLC16A2* gene groups, where low expression in each group resulted in improved OS compared to those with high expression [55].

A further study was assessed with the aim of highlighting the association of the clinical outcome of HNSCC patients with a molecular signature using transcriptomic data from 43 TCGA HNSCC samples as a discovery set. As a result, 886 differentially expressed genes were obtained between tumor tissue and adjacent normal tissue. Seven genes (*AATF*, *APP*, *GNPDA1*, *HPRT1*, *LASP1*, *P4HA1* and *ILF3*) were selected for biomarker analysis, all of which were significantly overexpressed in tumor tissue and significantly associated with OS. Patients with HNSCC were classified into low-risk and high-risk groups by defining the cutoff value (score = 0.36). The risk score was able to significantly distinguish the prognosis of 73 patients with HPV-positive HNSCC from a validation cohort (GSE65858) and 35 cases of HPV-positive HNSCC from TCGA, with the group of patients with the high-risk score being associated with unfavorable OS [56].

Importantly, in our literature review, an *NEFH* (neurofilament heavy chain) transcript was identified in two studies (Table 1). In one of them, *NEFH* is part of a molecular signature of eight high-risk genes for decreased survival in patients with HPV-positive HNSCC [45]; while in the second study, high *NEFH* expression was associated with better OS in HPV-positive HNSCC [50]. However, given that both studies have a limited number of samples and diverse analytical strategies, it cannot be assumed that NEFH is one of the most promising prognostic biomarker candidates in HPV-positive HNSCC, so further evidence to support this claim is required.

### 3.3. miRNAs

In a study conducted by Xiao-Jie Luo, et al., in 2021 [57], a total of 515 HNSCC samples and 44 adjacent normal tissue samples (control) with miRNA transcriptomic data were used, and further, the miRNAs differentially expressed were analyzed between 97 samples positive to HPV and 418 samples negative to HPV, where each cluster was compared with the control group. As a result, 282 and 289 miRNAs were differentially expressed in HPV-positive and -negative HNSCC cases, respectively. For each of the groups, a signature of miRNAs related to survival was identified and the risk score was calculated for each of the molecular signatures. The median risk score was selected as the cut-off point to separate the HPV-positive HNSCC group into high and low risk. In the case of miRNA signature associated with survival in HPV-related tumors, hsa-miR-380-5p, hsa-miR-493-3p, hsa-miR-454-5p, hsa-miR-376c-3p, hsa-miR-338-5p, hsa-miR-16-1-3p, and hsa-miR-378a-3p were found. Interestingly, it was also shown that this miRNA signature may serve as an independent predictor of the clinical stage. Furthermore, through gene set enrichment analysis (GSEA), target genes of the miRNA signature were associated with HPV-positive tumors mainly related to immune-enriched signaling pathways, suggesting that HPV-positive HNSCC patients with favorable prognosis could have better immune systems against tumors. Particularly, miRNAs associated with poor prognosis were found to be negatively correlated with CD8+ T cells. While miRNAs associated with good prognosis were positively correlated with activated NK cells and regulatory T cells [57].

In a study carried out by Cinzia Bersani et al., 2018 [58], a cohort of 162 cases of tonsil and base-of-tongue cancer (TSCC/BOTSCC) were analyzed, of which 110 and 52 were HPV-positive and -negative, respectively. It was observed that miR-155 expression is significantly lower in tumors than in normal tissue. Furthermore, elevated expression of miR-155 was strongly associated with high levels of CD8+ tumor infiltrating lymphocytes in HPV-positive TSCC/BOTSCC. Interestingly, high expression of miR-155 was shown to be significantly associated with increased OS and PFS of patients with HPV-positive tumors.

With the aim of developing and validating a prognostic signature based on miRNA sequencing analysis, Lui X., et al., 2021 [59] analyzed 324 OPSCC samples. As a result, 620 miRNAs were identified for subsequent analysis as prognostic biomarkers. Forty-five potential miRNAs were identified whose expression correlated with OS, of which only 40 retained their independent prognostic value after multivariate analysis. Twenty-six miRNAs with potential utility as biomarkers were retained for further signature development. Among these, 15 miRNAs (hsa-miR-361-3p, hsa-miR-374c-5p, hsa-miR-150-5p, hsa-miR-374b-5p, hsa-miR-107, hsa-miR-125b-5p, hsa-miR-1287-5p, hsa-miR-146a-5p, hsa-miR-106a-5p, hsa-miR-15b-5p, hsa-miR-20b-5p, hsa-miR-532-3p, hsa-miR-361-5p, hsa-miR-363-3p, hsa-miR-625-3p) and 11 miRNAs (hsa-miR-27a-3p, hsa-miR-455-5p, hsa-miR-203a-3p, hsa-miR-584-5p, hsa-miR-24-3p, hsa-miR-548k, hsa-miR-126-3p, hsa-miR-126-5p, hsa-miR-365a-5p, hsa-miR-98-5p, hsa-miR-151b) were associated with favorable and unfavorable outcomes, respectively. Patients were divided into high-risk- and low-risk-score groups. Survival analysis indicates that high- and low-risk groups exhibited markedly different risks in OS, as well as MFS and RFS. Furthermore, when the risk signature was evaluated exclusively in 280 HPV-positive OPSCC patients, the risk of OS and disease relapse (MFS and RFS) for the high-risk group was also increased compared with that for the low-risk group. These findings were validated in another cohort of 103 patients with HPV-positive OPSCC who were stratified into high- and low-risk groups according to the signature obtained previously. It was observed that the high-risk group exhibited significantly lower OS, MFS, and RFS compared to the low-risk group. These results demonstrate that the miRNA signature retained its prognostic significance when applied to independent patient cohorts [59]. 

## 4. Future Directions and Perspectives

### 4.1. Heterogeneity of HPV-Associated Biology as a Source of New Clinical Biomarkers

Massive next-generation sequencing and other omics technologies have revealed an unexpected level of heterogeneity that allows to describe emerging biomarkers to stratify patients with HPV-positive HNSCC. Understanding how HPV infection perturbs cancer genomes has highlighted the importance of HPV integration in clinical outcomes. HPV DNA integration into the host genome is a random process that leads to increased omics alterations at integration sites, resulting in activation of oncogenes and inactivation of tumor suppressors, triggering downstream effects that could alter tumor omics programs and the microenvironment. Based on clinical findings, it has been observed that high HPV integration correlates with worse survival [60,61], as it can enhance less sensitivity to standard therapies.

Given the important role of HPV integration in the development and progression of HNSCC, knowledge of the molecular landscape of viral integration and its biological consequences represents a link to clinical biomarker detection. For instance, the insertion of the HPV genome into the coding regions of the programmed death ligand 1 (*PDL1*) gene, resulting in its overexpression, has clinical significance as a useful prognostically protective biomarker in patients with HPV-positive tumors [62,63]. Whereas oncogenic viral integration favors overexpression of the *TP63* gene conferring aggressive phenotypes such as keratinization and cell adhesion allowing tumor cell survival, either by direct or indirect repression of proapoptotic genes. Exploiting the value of viral site of integration as a biomarker could allow to stratify patients at risk and identify those who could benefit from certain treatments, such as EGFR and PI3K inhibitors, along with radiotherapy [64,65]. In summary, transcriptional data are useful to better detect HPV integration or HPV episome maintenance, which should be considered during the diagnosis and treatment of HPV-positive tumors due to the differential prognostic outcomes of patients [61,66].

Currently, many patients with HPV-positive HNSCC are treated with high-dose radiation and chemotherapy. New biomarkers could recognize biologically distinct tumor subclasses of HPV-positive tumors with differential responses to radiotherapy to provide more biologically targeted treatment strategies with fewer side effects. Characterization of the transcriptional landscape of 104 HPV-positive HNSCC, together with public gene expression profiles, identified subclasses of HPV-positive carcinomas based on transcriptional programs or modules, independent of classical features such as loss of HPV E2, activation of PIK3CA programs, and APOBEC-mediated mutagenesis. Tumors with high expression of the NF-κB module have markedly better clinical outcomes, and preclinical characterization of this tumor class showed increased sensitivity to radiation [67]. Thus, accurate stratification of patients based on transcriptional biomarkers of non-canonical HPV drivers would allow better selection of treatment strategies and doses.

To better unravel the heterogeneity within HPV-positive tumors, a more sophisticated view using single-cell resolution was used to describe HPV-associated cellular phenotypes based on viral gene expression. This approach helped to identify the presence of a subset of HPV-positive cells with undetectable HPV expression (E6 and E7 oncoproteins), termed HPV-off and characterized by a phenotype of decreased proliferation and increased senescence, which are associated with decreased response to treatment, increased invasiveness, and poor prognosis, which could be attributed in part to augmented integration events. These findings suggest the existence of a group of HPV-positive malignant cells that lose an active transcriptional state, resulting in the suppression of hyperproliferative features, which ultimately confers resistance to conventional treatments for HPV-positive HNSCC tumors [68].

**Figure 1:**
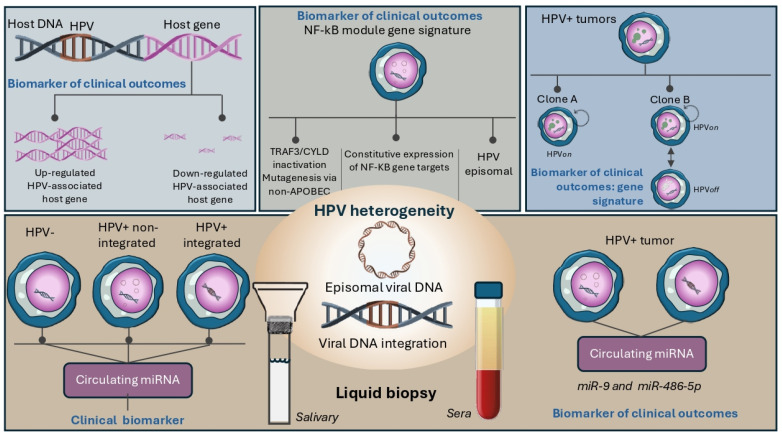
The heterogeneity of HPV-positive HNSCC requires an understanding of the molecular biology underlying the abundance in the expression of viral proteins (HPV-on or HPV-off) or events generated by the integration of the viral genome into the cellular genome. Moreover, accurate stratification of patients based on transcriptional biomarkers such as NF-κB and APOBEC genes, dependent on non-canonical HPV drivers, would allow better selection of treatment strategies. Recently, liquid biopsy has been represented as a promising methodological tool for the identification of biomolecules such as circulating miRNAs associated with the clinical outcome of patients with HPV-positive HNSCC.

The application of analytical enrichment methods to infer the presence of these modules or phenotypes through transcriptomic signatures would provide new biomarkers to stratify HPV-positive tumor subtypes. Despite advances in the development of biomarkers for HPV-positive HNSCC tumors, some challenges remain. Understanding biological principles and the use of high-throughput technologies can help identify relevant biological alterations in HPV-positive tumors. In Figure 1, biological gene expression alterations are depicted to illustrate the potential role of tumor biomarkers based on tumor heterogeneity in HPV-positive HNSCC prognosis and therapy.

### 4.2. Unraveling Tumor Biology by HPV-Associated Circulating miRNAs

Multiple approaches for tumor biomarker detection have been developed in the last decade. In recent years, liquid biopsy has attracted much attention and miRNAs represent a promising class of biomolecules for this application, as they offer advantages in terms of stability, abundance and specificity. The comprehensive evaluation of circulating miRNAs in various biofluids opened new windows for the identification of HPV-positive tumors (Figure 1). For example, salivary miR-122, miR-124, miR-205 and miR-146a are potential differentiators between HPV-positive and HPV-negative cancers [69]. Other studies aimed at differentiating HPV-positive and HPV-negative patients reported the discriminatory value of salivary miR-9, miR-134, miR-196b, miR-210 and miR-455 [70], as well as salivary miR-486-5p and miR-20-5p isolated in exosomes of both head and neck cancers and HPV-positive/p16 cervical cancers [71,72]. These data provide clear evidence for the existence of novel circulating molecules that allow to discriminate between HPV-associated tumors. Moreover, some of the miRNAs identified by liquid biopsy have also been associated with clinical outcomes. For example, increased miR-9 sera levels are positively associated with better prognosis in patients with oral squamous cell carcinoma [73]. Circulating miR-486-5p is also a surrogate marker of postsurgical recurrence, as its differential expressions in paired pre- and postsurgical plasma samples are associated with recurrence [74].

The use of liquid biopsies in oncology has demonstrated their potential to inform disease prognosis, progression and responses to therapies. Several transcriptomic biomarkers have been identified that can be assessed in HPV-positive HNSCC tumors. It should be noted that the sensitivity and specificity of assays in multiple biofluids and head and neck cancers pose some challenges. However, with improving technology and analytical approaches to integrate multiple molecules and analytes, we anticipate that liquid biopsy will soon improve clinical outcomes in this neoplasia.

## 5. Concluding Remarks

The use of genomic technologies has allowed the identification of several molecules highly related to the development and maintenance of human neoplasms, in addition to providing relevant information for the development of new pharmacological strategies. The detailed molecular characterization of HNSCC through massive nucleic acid sequencing and analysis of open-access repositories with diverse transcriptomic data suggests that the incorporation of prognostic biomarkers in the clinical management of patients could overcome, in the near future, the current obstacles to targeted therapies and have an impact on clinical outcome of patients.

The study of the molecular processes underlying the presence of HPV in HNSCC tumors, such as the identification of gene expression profiles, will allow a greater understanding of tumor heterogeneity, offering a great opportunity to discover molecular drug targets and identify a group of patients with HPV-positive HNSCC who are at risk of relapse or recurrence, and also a group of patients who could benefit from dose de-escalation treatments, obtaining equal or better results, with a reduction in side effects. Currently, it is of interest to analyze the expression of several transcripts in HPV-positive HNSCC that, combined with the clinicopathological characteristics of the patients, will permit proposing biomarkers with prognostic value and designing therapeutic strategies aimed at improving the quality of life of patients. Each of the studies included and discussed in this manuscript propose the potential use of prognostic biomarkers in a group of head and neck tumors, particularly those positive for HPV infection, which until now have been considered to have a good prognosis compared to HPV-negative cases. However, more than 30% of these patients experience recurrence in less than 5 years, which evidences that even within the group of HPV-positive tumors, there is another molecular subgroup associated with an unfavorable clinical outcome. Several bioinformatic analyses have been aimed at the discovery of transcripts with potential use as biomarkers, and new workflows and stricter algorithms constantly emerge that allow the analyses to be strengthened. In addition, it is imperative to apply these workflows to other validation cohorts, not only those available in databases from repositories but also in the populations where it is desired to test the proposed biomarkers, considering the intrinsic characteristics of each population.

Certainly, when using omics data, it is desirable to achieve high replicability and reproducibility to obtain consistent quality of results as well as compatible quantitative data between studies. Multiple variables that affect replicability have been identified, such as large differences in the characteristics of the analyzed samples, reduced sample sizes, variations in analysis methods and more complex data distribution properties, among others. In general, low replicability and significant variations between results have been observed in studies using TCGA data [75]. This is why it is necessary to emphasize the development of tools that allow examining multiple measures of replicability and studying their distributional properties to increase the robustness and reliability of results obtained from omics data.

Although all transcript-based biomarkers discussed in this review are valuable, the high variability of the results makes it currently difficult to suggest the best candidate as a prognostic biomarker; therefore, the proposed transcripts or their protein products need to be validated in independent cohorts through gold standard methods such as PCR, immunohistochemistry or FISH, which have demonstrated utility and reproducibility.

## Figures and Tables

**Table 1:** Biomolecules significantly associated with the clinical outcome of HPV-related HNSCC patients.

Name	Level	Biomolecule	Cancer Type	Clinical Outcome Impact	References
*PRINS* (Expression)	High	lncRNA	HNSCC	Improved OS and DFS	[43]
*CD3E*, *IRF4*, *ZAP70* (UWO3 Risk score)	Low	mRNA	OPSCC	Improved OS and DFS	[44]
*Clorf105*, *CGA*, *CHRNA2*, *CRIP3*, *CTAG2*, *ENPP6*, *NEFH*, *RNF212* (Risk score)	High	mRNA	HNSCC	Worse OS and DFS	[45]
*S100A9* *THBS4* (Expression)	Low High	mRNA	OPSCC	Worse MFS	[46]
*NRF2* *NQO1* *TP53* *KEAP1* (Expression)	Low Low Low High	mRNA	HNSCC	Improved OS	[47]
m*CBX5*, m*CDKN2A*, m*MCM5*, m*MCM6*, m*RBBP7*, m*TMPO*, m*VCAM1* (Risk score)	High	mRNA	HNSCC	Worse OS	[48]
*ZNF540* (Expression)	High	mRNA	HNSCC	Improved OS	[49]
*FDCSP* *NEFH* *FAM3B* (Expression)	High High High	mRNA	HNSCC	Improved OS	[50]
*FDCSP* (Expression) CD8+ T cells (Infiltration)	High High	mRNA	HNSCC	Improved OS	[50]
*U62317.3* hsa-miR-375 *KLHD* (Expression)	High Low High	lncRNA miRNA mRNA	HNSCC	Improved OS	[51]
*PDLIM5* *USP25* *SLMAP* (Expression)	Low Low Low	mRNA	HNSCC	Worse OS	[51]
*SLC25A39* *GJB2* (Expression)	High High	mRNA	HNSCC	Worse OS	[52]
*DOCK8* (Expression)	High	mRNA	HNSCC	Improved OS and DFS	[53]
*RASSF8*, *LGALSL*, *TRIB3*, *FAM106A*, *MICAL3*, *STIP1*, *ZNF146*, *ZNF284*, *TPST1*, *PSMG3*, *CPEB2*, *SH3D21*, *CNOT2*, *ENTPD6*, *PPIAL4C*, *SARS*, *SULT1E1*, *EAF1*, *LOC100128108*, *PLPPR2*, *OTUD4*, *MUL1*, *BCL2L13*, *GUCY1B1*, *MRPL45*, *GALK1*, *RSU1*, *AVPI1*, *PDPR*, *ALKBH6*, *HCK*, *ATP6V1A*, *TAF5L*, *BUD13*, *TNFRSF6B*, *ACOT11*, *RNF167*, *ORAI1*, *CRYBG3*, *HDDC3*, *CREB3L4*, *TMEM246*, *PEX16*, *HAUS1*, *NUP214*, *AURKB*, *OGN*, *FBXO41*, *SLFN13*, *CXCL13*, *COMMD3*, *FOXRED2*, *FPGS*, *UCP2*, *GLUL*, *LYN*, *MEI1*, *CYBA*, *NUP210*, *ARHGAP4* (Risk score)	Low	mRNA	OPSCC	Improved OS, RFS and MFS	[54]
*SDHC* *COX7A1* *COX16* *COX17* *ELOVL6* *GOT2* *SLC16A2* (Expression)	Low Low Low Low Low Low Low	mRNA	HNSCC	Improved OS	[55]
*AATF*, *APP*, *GNPDA1*, *HPRT1*, *LASP1*, *P4HA1*, *ILF3* (Risk score)	High	mRNA	HNSCC	Worse OS	[56]
hsa-miR-380-5p, hsa-miR-493-3p, hsa-miR-454-5p, hsa-miR-376c-3p, hsa-miR-338-5p, hsa-miR-16-1-3p, hsa-miR-378a-3p (Risk score)	Low	miRNA	HNSCC	Improved OS	[57]
miR-155 (Expression)	High	miRNA	TSCC/BOTSCC	Improved OS and PFS	[58]
hsa-miR-27a-3p, hsa-miR-455-5p, hsa-miR-203a-3p, hsa-miR-584-5p, hsa-miR-24-3p, hsa-miR-548k, hsa-miR-126-3p, hsa-miR-126-5p, hsa-miR-365a-5p, hsa-miR-98-5p, hsa-miR-151b, hsa-miR-361-3p, hsa-miR-374c-5p, hsa-miR-150-5p, hsa-miR-374b-5p, hsa-miR-107, hsa-miR-125b-5p, hsa-miR-1287-5p, hsa-miR-146a-5p, hsa-miR-106a-5p, hsa-miR-15b-5p, hsa-miR-20b-5p, hsa-miR-532-3p, hsa-miR-361-5p, hsa-miR-363-3p, hsa-miR-625-3p (Risk score)	High	miRNA	OPSCC	Worse OS, RFS and MFS	[59]

HNSCC: Head and Neck Squamous Cell Carcinoma; OPSCC, Oropharyngeal Squamous Cell Carcinoma; TSCC: Tonsil Squamous Cell Carcinoma; BOTSCC: Base of Tongue Squamous Cell Carcinoma; OS: Overall Survival; DFS: Disease-Free Survival; MFS: Metastasis-Free Survival; RFS: Relapse-Free Survival; PFS: Progression-Free Survival.
